# Prevalence of wasting and associated factors among children aged 6-59 months in Wolkite town of the Gurage zone, Southern Ethiopia, 2020. A cross-sectional study

**DOI:** 10.1371/journal.pone.0259722

**Published:** 2022-01-27

**Authors:** Alex Yeshaneh, Tinsaye Mulu, Addisu Gasheneit, Daniel Adane

**Affiliations:** 1 Department of Midwifery, College of Medicine and Health Sciences, Wolkite University, Wolkite, Ethiopia; 2 Awi Zone Health Office, Amhara Regional State Health Bureau, Bahir Dar, Ethiopia; 3 Department of Midwifery, College of Health Sciences, Injibara University, Injibara, Ethiopia; Federal University of Sergipe, BRAZIL

## Abstract

**Background:**

Undernutrition among children is one of the leading major public health problems and about 49.5 million children were wasted worldwide. Asia and African countries contributed 69% and 27.2% of wasting respectively. In Ethiopia, 7% of children were wasted and 1% was severely wasted. Although Ethiopia has achieved remarkable progress in reducing under-five mortality and designed multi-dimensional approaches to address malnutrition, the data on acute malnutrition among children in the study setting is limited. Therefore, this study was aimed to assess the prevalence of acute malnutrition and associated factors among 6-59-month-old children.

**Methods:**

Community-based cross-sectional study design was conducted at Gurage Zone, Southern Ethiopia. A total of 293 study participants were selected using a systematic sampling technique. Data were collected using structured and pre-tested interviewer-administered questionnaires by face-to-face interview. Data entry and analysis were made using Epi Data version 4.6 and Statistical Package for Social Science (SPSS) version 24 respectively. Descriptive statistical analysis and some of the statistical tests like the odds ratio were carried out. Both Bivariable and multivariable logistic regression analysis with 95% confidence interval was carried out to identify associated factors and variables with P value < 0.05 were taken as statistically significant.

**Results:**

The prevalence of wasting among children aged from 6–59 months in this study was 14.7% (95% CI: 10.9, 18.8). After controlling for all possible confounding factors, the result revealed that age of children between 6–11 months [AOR = 2.78(95% CI: 1.67, 6.19)], caregivers who were unable to read and write [AOR = 2.23 (95% CI: 1.04, 5.34)], presence of diarrheal disease in the past two weeks [AOR = 1.68 (95% CI: 1.23, 5.89)] and mothers who had a history of poor handwashing practice before food preparation and child feeding [AOR = 2.64(95% CI: 1.52, 4.88)] were found to be significantly associated with wasting.

**Conclusions:**

The study findings indicate that respondents’ wasting was mainly affected by age of the child, educational status of caregivers, presence of diarrheal disease and hand washing practice of the mother. Providing acceptable, quality and honorable care for all children is very crucial to prevent child wasting and proper handwashing during breastfeeding and food handling is recommended and interventions aimed at improving maternal health and access to health care services for children are urgently needed.

## Background

Malnutrition results from eating a diet in which one or more nutrients are either not enough or are too much [1]. Malnutrition is often used specifically to refer to undernutrition where an individual is not getting enough calories, protein, carbohydrates, or micronutrients [2]. In developing countries, undernourishment is often due to a shortage of high-quality food secondary to high food prices and poverty [3]. It is classified into chronic and acute undernutrition. Acute undernutrition (Wasting) is the result of inadequate food intake usually from a recent episode of illness that caused weight loss and is also characterized by a rapid deterioration in nutritional status over a short period in under-five children [4, 5].

In children, wasting can be measured using the weight-for-height nutritional index or mid-upper arm circumference (MUAC). There are different levels of severity of acute malnutrition: Moderate acute malnutrition (MAM) is defined by a weight-for-height index between -3 and less than -2 Z-score or between 11.5 to <12.5 cm by MUAC. Severe acute malnutrition (SAM) is the most dangerous form and if left untreated, it can result in death [6, 7].

Undernutrition among children is one of the leading major public health problems throughout the world. In 2020, 1 in 9 people were hungry or undernourished and about 49.5 million children under five were wasted worldwide. Asia and African countries contributed 69% and 27.2% of wasting respectively [8]. Among all under-five child deaths, childhood malnutrition was responsible for 35% of deaths. More than 2 million children die each year as a result of undernutrition before the age of 5 years [9]. In Ethiopia, 7% of children were wasted, and 1% was severely wasted [10].

Undernourished children are physically, emotionally and intellectually less productive and they will be suffered from chronic illnesses and disabilities. This, in turn, increases health care costs, reduces productivity and slows economic growth, which can perpetuate the cycle of poverty and ill-health [11, 12]. In Ethiopia, the prevalence of wasting was 10% in EDHS, 2016 [4] and 7% in Ethiopia mini demographic and health survey (EMDHS) [10]. There was also the regional variations with the highest percentages of wasting in under-five children such as 21% in Somali, 14% in Afar, 13% in Gambella, 2% in Addis Ababa and 4% in Harari [10], 13.4% in Bulehora district [13], 10% in Northwest Ethiopia [14], 11% in Adi-Harush and Hitsats Refugee Camps in Tigray Region [15], 11.1% in Dilla town [16] and 28.2% in Hawassa Zuria district [17].

The socio-demographic, child-caring practices, environmental health and sanitation-related factors are the most prominent risk factors that determine acute undernutrition among under-five children [15–17]

Combating undernutrition in all its forms is one of the greatest global health challenges [8] and evidence-based interventions to overcome undernutrition are mostly recommended. Although Ethiopia has already achieved remarkable progress in reducing under-five mortality in the last decades and designed multi-sectorial and multi-dimensional approaches to address malnutrition, the data on undernutrition among 6–59 months old children of the study setting is limited [7, 18, 19]. Therefore, this study was aimed to assess the prevalence of acute malnutrition and associated factors among children aged between 6 and 59 months in Wolkite town, southern Ethiopia.

## Methods and materials

### Study setting, design and population

Community-based cross-sectional study design was conducted from December to January 2020/2021 in Wolkite town, Southern Ethiopia. Wolkite is the administrative center of the zone and is found 158 kilometers far from the capital city (Addis Ababa) in the Southern region of Ethiopia. It has a total population of 70,796 people of these 53% were males and 47% were females. The proportions of the under-five population were 2,169 populations. The town has five Kebele (Menaheriya, Edigetchora, Selamber, Adishiwot, and Edigetber). The total populations of the two Kebele (Menaheriya and Edigetber) were 13,359 with a total of 2,618 households. The source populations of this study were all 6–59 months old children (paired with their mothers or caregivers) whereas all randomly selected 6–59 months old children (paired with their mothers or caregivers) during the study period were considered as the study population.

### Eligibility criteria

Mothers who reside for at least six months in the study area having a child aged 6–59 months were included in the study. Whereas, children with evidence of physical impairment, seriously ill, mentally impaired and those mothers’/caregivers who were unable to communicate were excluded.

### Sample size determination

The sample size was determined using the single population proportion formula by considering the following assumptions; Proportion of prevalence of acute undernutrition (wasting) of 28.2% (P = 0.282) [17], 95% confidence level, the margin error of 5% (d = 0.05). The sample size for the second objective (factors) was also determined by using the double population proportion formula for cross-sectional study by considering the following assumptions as Power = 80%, CI = 95% and Ratio = 1:1. The largest sample size from those samples was taken and the final sample size was calculated to be 311 children paired with their mother/caregivers.

### Sampling technique

From five Kebele of the town, 2 Kebele ((Meneheriya (1458 households) and Edigetber (1160 households)) were selected randomly. To select study participants systematic sampling technique was used. Those eligible participants who did not avail themselves during the data collection period in selected Kebele were revisited three times and if not avail after three visits the data collector skip that house and interview the next household to substitute. To select a total of 311 respondent’s proportional allocation to population size was used in each Kebele. The first participant was selected randomly and every 8^th^ participant who had under-five children were interviewed.

#### Dependent variable

Wasting (Yes/No)

#### Independent variables

*Sociodemographic variables*. Child age, Child sex, maternal educational status, maternal occupation, Family size, Religion, Ethnicity, Household food security.

*Child caring practice and health characteristics*. Exclusive breastfeeding, Dietary diversity score, Meal frequency, Vaccination status, History of diarrheal in the past two weeks, ever used family planning, Place of delivery.

*Environmental health-related variables*. Availability of latrine, Hand washing practice, Solid waste disposal, Availability of liquid waste disposal pit.

### Operational definitions

#### Wasting

The child weight-for-height Z-score (WHZ) is <-2 SD from the median WHO reference values is wasting, WHZ ≥ −3SD & < −2SD is Moderate Wasting and WHZ < −3SD is Severe wasting [20].

#### Undernutrition

MUAC below 12.5 cm indicates acute undernutrition, MUAC ≥ 11.5 cm & < 12.5 cm indicates moderate acute undernutrition and MUAC < 11.5 cm indicates severe acute undernutrition [20].

#### Diarrhea

Defined as having three or more loose of watery stools in 24 hours in the two weeks before the survey [21].

#### Complementary food

Foods that are required by the child, after six months of age in addition to sustained breastfeeding [1].

#### Household food security

Measured whether the respondent worries that the household would not lack have enough food for the past four weeks [2].

#### Food secure

If the respondent does not worry that the household would lack enough food for the past weeks [2].

#### Mildly food insecure

Rarely worry about food (once or twice in the past four weeks) [2].

#### Moderately food insecure

Sometimes worry about food (three to ten times in the past four weeks) [2].

#### Severely food insecure

Often (more than ten times in the past four weeks) [2].

#### Good handwashing practice

If the respondent washes hand before and/ or after actions (before cooking, before eating, after latrine visit, after child cleaning, before child feeding…) [3].

#### Continued breastfeeding at 1 year

Children 12–15 months of age who continued breastfeeding after the age of 1 year [3].

#### Continued breastfeeding at 2 years

Children 20–23 months of age who continued breastfeeding after his/ her 23 months of age [3].

### Data collection tools and procedure

An English version semi-structured interviewer-administered questionnaire was developed by reviewing different works of literature, current national and international guidelines of child nutrition. The tool consisted of socio-demographic characteristics, child-caring practice and health characteristics and environmental health-related characteristics of respondents. Ten Bachelors of Science in midwives and one Master of Science in midwife were recruited to support data collection. Recumbent length was assessed for all children under 24 months of age while standing height was measured for older children. Children were weighed having lightly clothing, without shoes and with empty pockets. Mid Upper Arm Circumference (MUAC) was measured using non-stretchable tape on the left mid-upper arm to the nearest 1 mm.

### Data quality assurance

One day of training was given for data collectors and supervisors on objectives and the standard procedures of MUAC measurement. A height measuring length board that has a scale and sliding headpiece and a 2 meters measuring capacity, with a precision of 0.1 cm was used for measuring the height. UNICEF’s digital weighing scale (SECA) which has a capacity of 150 kg and with a precision of 0.1 kg was used for measuring weight. By considering 5% of the total sample size pretest was conducted one week before the start of actual data collection in the Endiber town which was not part of this study. Then the questionnaire was assessed for its clarity, length, completeness and the necessary correction was done accordingly. Throughout the data collection, interviewers were supervised, regular meetings were held between the data collectors and the principal investigator together in which problematic issues arising from interviews during the data collection and any challenges found were discussed. The completeness of the data was evaluated by field supervisors daily. The collected data were again reviewed and checked for its completeness before data entry. The data entry format template was prepared and programmed by the investigators.

### Data processing and analysis

After data collection was completed, the data were checked for completeness and then recoding and categorization were done. Data entry and analysis were done using Epi Data version 4.6 and SPSS version 24 respectively. Software program WHO AnthroPlus was used to convert nutritional data from anthropometric measurement into Z-score of the indices: weight for height, considering sex using WHO reference curves. WHZ was calculated for each child using the WHO growth reference standards and WHZ <-2 SD is categorized as wasting [20]. Descriptive statistical analysis was carried out to identify frequency, percentage and mean for continuous independent variables. Before the analysis, the assumptions of the chi-square test were checked. Binary logistic regression analysis was used to ascertain the association between the dependent and independent variables. Variables with a significant association at P < 0.2 in the binary analysis were entered into multivariable analysis using the enter method to determine the factors associated with wasting and those variables P<0.05 were considered to be statistically significant. Hosmer- Lemeshow tests for goodness of fit were carried out and found p = 0.889(>0.05). A Multi-collinearity test was carried out to see the correlation between independent variables by using collinearity statistics (Variance inflation factor (VIF) >10 and standard error >2 was considered as suggestive of the existence of multi co-linearity). Finally, the results were presented in texts, tables and graphs and it was discussed using the odds ratio and 95% confidence interval.

### Ethical approval and consent to participate

Ethical clearance was obtained from the Research and Ethical Review Committee of Wolkite University, College of Medicine and Health sciences. Permission to conduct the study was also obtained from Gurage Zone administrative office. The study purpose, procedure, duration, rights of the respondents and data safety issues, possible risks and benefits of the study were clearly explained to each participant using the local language. Then before the commencement of the study, all subjects gave their informed written consent. Participation in this study was purely voluntary and there was no monetary gain. No compensation was offered for participation in the study. All the participants’ response was kept confidential by using the information only for the study and storing the study in a closed file.

## Results

### Socio-demographic characteristics of the participants

In this study, a total of 293 respondents have participated with a response rate of 94.2%. The mean age and standard deviation (SD) of the children and the mother/caregivers were 27.48 ± 14.48 months and 28.97±4.57 years old respectively. Nearly half (50.5%) of the children were females and 21.5% of children were in the age group of 6–11 months. One hundred twenty-one (41.3%) were orthodox Christian religion followers. About 34.1% of the respondents had formal education up to the level of primary and 70.3% of the respondents have been working as a housewife (Table 1).

**Table 1:** Sociodemographic characteristics of study participants in Wolkite town, 2020/2021.

Variables (n = 293)	Categories	Frequency	Percent (%)
**Child age**	6–11 months	63	21.5
	12–23 months	50	17.1
	24–35 months	49	16.7
	36–47 months	65	22.2
	48–59 months	66	22.5
**Child sex**	Male	145	49.5
	Female	148	50.5
**Educational status of the mother**	Unable to read and write	21	7.2
	Able to read and write	48	16.4
	Primary education	100	34.1
	Secondary education	84	28.7
	College and above	40	13.7
**Occupation of the mother**	Housewife	206	70.3
	Government employee	20	6.8
	Merchant	55	18.8
	Private employee	4	1.4
	Daily laborer	8	2.7
**Family size**	1–4	101	34.5
	>4	192	65.5
**Religion**	Muslim	119	40.6
	Orthodox	121	41.3
	Protestant	42	14.3
	Others*	11	3.8
**Ethnicity**	Gurage	215	73.4
	Amhara	27	9.2
	Oromo	21	7.2
	Wolaita	19	6.5
	Others**	11	3.8
**Household food security**	Food secured	135	46.1
	Mildly food insecure	55	18.8
	Moderately food insecure	61	20.8
	Severely food insecure	42	14.3

Others*; Catholic and Adventist,

Others**; Tigre, Sidama, Afar

### Child caring practice and health

About 17.4% of respondents reported that they were started complementary feeding after the age of six months. Nearly two-third of households consume more than 4 types of food groups, but the majority, 68.9% of respondents consume less than three times per day. About 44.4% of respondents were fully vaccinated but 5.5% were not vaccinated. Sixty-four (21.8%) of the study participants have a history of diarrhea in the past two weeks before data collection (Table 2).

**Table 2:** Child caring practice and health characteristics of the study participants in Wolkite town, 2020/2021.

Variables (n = 293)	Categories	Frequency	Percent (%)
**Exclusive breastfeeding**	≤6 months	242	82.6
	>6 months	51	17.4
**Dietary diversity score**	≥ 4 foods	183	62.5
	< 4 foods	110	37.5
**Meal frequency**	<3/day	202	68.9
	≥3/day	91	31.1
**Vaccination status**	Not vaccinated	16	5.5
	Partially vaccinated	23	7.8
	Vaccinated	124	42.3
	Fully vaccinated	130	44.4
**History of diarrheal in the past two weeks**	Yes	64	21.8
	No	229	78.2
**History of using family planning**	Yes	290	99.0
	No	3	1.0
**Place of delivery**	Home	44	15.0
	Health facility	249	85.0

### Environmental health-related characteristics

The findings of this result revealed that almost all (99.3%) of the respondents have a latrine. Nearly 10% of respondents reported that they have poor handwashing practice and about 62.8% of the respondents have no liquid waste disposal pit (Table 3).

**Table 3:** Environmental health-related characteristics of study participants in Wolkite town, 2020/2021.

Variables (n = 293)	Categories	Frequency	Percent (%)
**Availability of latrine**	Yes	291	99.3
	No	2	0.7
**Hand washing practice**	Good	264	90.1
	Poor	29	9.9
**Solid waste disposal**	Pit	62	21.2
	Open	69	23.5
	Bag	55	18.8
	Municipality service	99	33.8
	Other	8	2.7
**Availability of liquid waste disposal pit**	Yes	109	37.2
	No	184	62.8

### Prevalence of wasting

**Fig 1:**
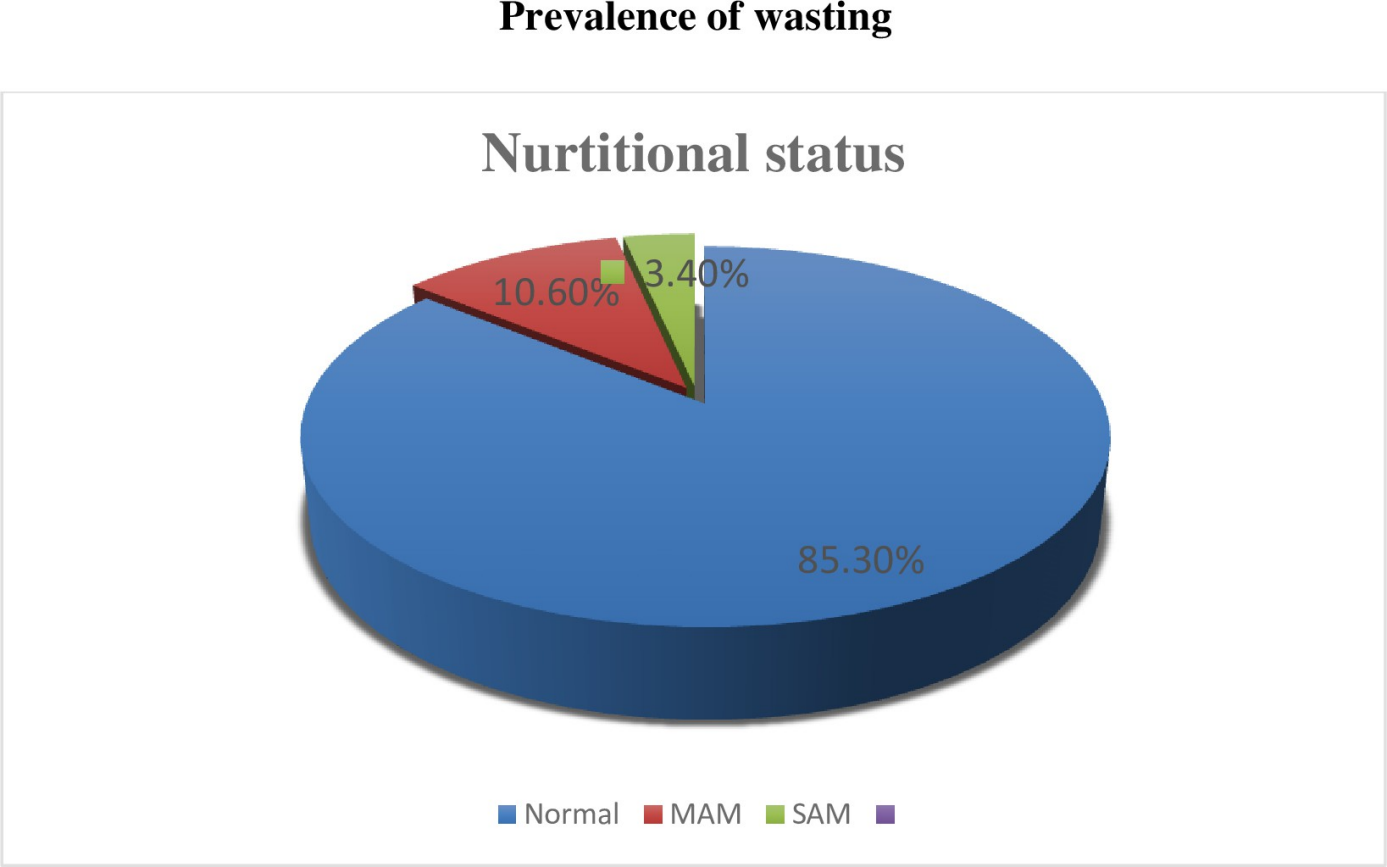
Prevalence of wasting among children from 6–59 months, in Wolkite Town, Southern Ethiopia, 2020/2021.

The finding of this study revealed that 14.7% of children were wasted (95% CI: 10.9, 18.8). Among these, 10.6% and 3.4% of them were grouped under moderate acute malnutrition (MAM) and severe acute malnutrition (SAM) respectively (Fig 1).

### Factors associated with wasting

The result of the Bivariable analyses showed that sex, birth order, maternal education, economic status of the family, and poor hand washing practice of caregivers were candidate variables for multivariable logistic regression analysis at a p-value of less than 0.2. The result of the multivariable analysis showed that children aged 6–11 months, mothers/caregivers who were not able to write and write in their education, recent diarrheal diseases and maternal poor hand washing practice before food preparation and child feeding were the factors associated with wasting at a P-value <0.05.

The odds of wasting were 2.78 [AOR = 2.78(95% CI: 1.67, 6.19)] times higher among children who were grouped at the age of 6–11 months than their counterparts. The odds of wasting were 2.23 [AOR = 2.23 (95% CI: 1.04, 5.34)] times higher among uneducated mothers/unable to read and write/ than those who were born from mothers who had an educational level of college and above. The odds of wasting were 1.68 [AOR = 1.68 (95% CI: 1.23, 5.89)] time higher among children who had a history of recent diarrheal diseases (two weeks before the data collection) than those who had no history of diarrhea. The odds of wasting were 2.64[AOR = 2.64(95% CI: 1.52, 4.88)] times higher among children born from mothers/caregivers who had a history of poor handwashing practice before food preparation and child feeding than their counterparts (**Table** 4).

**Table 4:** Factors associated with child wasting in Wolkite town, Gurage zone, southern Ethiopia, 2020/2021.

Variables (n = 293)	Category	Wasting			
		Yes	No	COR (95%CI)	AOR(95%CI)
Child age (months)	6–11	16	47	0.19(0.06–0.60)*	2.78(1.67–6.19)*
	12–23	10	40	0.26(0.08–0.88)*	0.28 (0.12–2.73)
	24–35	7	42	0.39(0.11–1.41)	1.56 (0.15–3.25)
	36–47	6	59	0.63(0.17–2.36)	1.14(0.59–4.85)
	48–59	4	62	1	1
Child sex	Male	17	128	1	1
	Female	26	122	0.62(0.32–1.21)	0.78(0.56–3.23)
Birth order	1^st^	8	39	1	1
	2–4	34	172	1.04(0.45–2.42)	0.80(0.56–1.98)
	>4	1	39	8.00(0.96–12.03)	4.04(0.45–6.44)
Education status of the mother	Unable to read & write	16	5	0.03(0.01–0.012)*	2.23(1.04–5.34)**
	Able to read and write	6	42	0.57(0.13–2.43)	0.61(0.35–3.67)
	Primary education	12	88	0.60(0.158–2.23)	0.87(0.12–1.84)
	Secondary education	6	78	1.05(0.250–4.45)	0.57(0.65–2.59)
	College and above	3	37	1	1
Household food security	Food secured	6	129	1	1
	Mildly food insecure	8	47	0.27(0.09–0.823)*	0.73(0.83–3.50)
	Moderately food insecure	20	41	0.09(0.04–0.25)*	0.48(0.15–3.24)
	Severely food insecure	9	33	0.17(0.06–0.51)*	2.32(0.87–4.34)
Duration of exclusive breastfeeding	<6 months	31	211	2.09(0.99–4.43)	1.26(0.12–3.76)
	>6 months	12	39	1	1
Diarrheal disease in the past two weeks	Yes	28	36	0.09(0.04–0.19)*	1.68(1.23–5.89)*
	No	15	214	1	1
Hand washing practice before food preparation and child feeding	Good	22	242	1	1
	Poor	21	8	0.04(0.01–0.09)*	2.64(1.52–4.88)**

*p <0.05,

**P<0.01

## Discussion

This study was conducted to assess the prevalence of wasting and associated factors among children aged between 6 and 59 months in Wolkite town, southern Ethiopia.

The finding of this study revealed that 14.7% of children were wasted. This finding is higher than the findings in 2019 Ethiopian Mini Demographic and Health survey result (7%) [10], Northwest Ethiopia (10%) [14] and Adi-Harush and Hitsats Refugee Camps in Tigray (11%) [15]. The difference might be due to the difference in the study period and the population participated in the study. This finding is slightly higher than the study done in Dilla town (11.1%) [16]. This differences may be because of the difference in population participated in the study as the former study focused on under-five orphans which implies more emphasis might be given by different stakeholders to prevent wasting. On the other hand, the finding of this study is lower than the study conducted at Hawassa Zuria, South Ethiopia (28.20%) [17]. This might be due to the difference in the study population; the study in Hawassa was implemented for the age group of 36–60 months but this study was conducted among those children of 6–59 months. Other possibilities might be due to the difference in the study area; the former study was conducted in the rural areas where that had limited access to health facilities, information and supply of foods than urban areas.

In this study, we have found some factors associated with wasting. These include children age 6–11 months, uneducated mother/caregiver, having a history of diarrheal disease in the past two weeks and a history of poor maternal handwashing practice before food preparation and child feeding. The odds of wasting among children who were grouped at the age of 6–11 months were 2.78 times higher than their counterparts. This study is supported by the study conducted in Tanzania [22], Northwest Ethiopia [14]. This is because those children who are born from poor families (African families) might have inadequate quality or quantity of feeding practice which implies they are prone to undernutrition that can lead to poor cognitive and motor development.

The odds of wasting were 2.23 times higher among uneducated mothers than those who were born from mothers who had an educational level of college and above. This finding is consistent with the study conducted in Northwest Ethiopia [14]. This might be explained as the mother/caregiver who did not attend formal education might have poor understanding and limited access to information about the advantage of child nutrition as compared to educated caregivers.

The odds of wasting were 1.68 times higher among children who had a history of recent diarrheal diseases (two weeks before the data collection) than those who had no history of diarrhea. This study was supported by the study done in the Bulehora district of South Ethiopia [13] and Karat town of Southern Ethiopia [23]. This is because those children who had diarrhea will have often been prone to rapid weight loss and hence they may become wasted.

The odds of wasting were 2.64 times higher among children born from mothers/caregivers who had a history of poor handwashing practice before food preparation and child feeding than their counterparts. The finding of this study is supported by the study conducted in Northwest Ethiopia [14]. This is evidenced as those children who were born and live in poor hygienic areas might have diarrhea which may affect nutritional status and finally, it might lead the child to be wasted.

### Limitation of the study

This study shares the limitation of the study design. It does not include those children who had physical/mental disability and children aged below 5 months and above 59 months, therefore this study did not generalizable for all children rather for those age groups who are in between 5–59 months old.

## Conclusion

The prevalence of wasting among children aged from 6–59 months in the town was 14.7%. The age group of children between 6–11 months, uneducated mother/caregiver, history of diarrheal disease in the past two weeks and poor handwashing practice before food preparation and child feeding of the mothers was factors associated with wasting. Wolkite town health bureau and other stakeholders should work towards nutrition-related programs to overcome the problem of wasting and its associated factors at a community level. Health extensions workers should be collaborated with others sectors and stakeholders to improve the knowledge of mothers’ child care practice. Nutrition surveillance should be done continuously and special attention should be given to vulnerable groups such as the poorest and the most severely malnourished children.

## List of abbreviations

AOR: Adjusted Odds Ratio
CI: Confidence Interval
COR: Crudes Odds Ratio
MAM: Moderate Acute Malnutrition
MUAC: Mid Upper Arm Circumference
SAM: Severe Acute Malnutrition
SPSS: Statistical Package for Social Sciences
UNICEF: United Nations International Children’s Emergency Fund
